# Changes in the provision of psychiatric beds and incarceration in the Eastern Mediterranean Region from 1990 to 2022

**DOI:** 10.3389/fpsyt.2025.1539662

**Published:** 2025-07-16

**Authors:** Adrian P. Mundt, Mohammad Akbar Paiman, Heyam Dalky, Joseph El-Khoury, Suhaila Ghuloum, Mohammed Mohammed, Sabine Delhey-Langerfeldt, Feten Fekih-Romdhane, Medhat Elsabbahy, Mohammad Marie, Maan Saleh, Stefan Priebe, Enzo Rozas-Serri

**Affiliations:** ^1^ Centro de Investigación Biomédica, Medical Faculty, Universidad Diego Portales, Santiago, Chile; ^2^ Facultad de Medicina Norte, Hospital Clínico Universidad de Chile, Santiago, Chile; ^3^ Epidemiology, Ministry of Public Health, Kabul, Afghanistan; ^4^ Nigeria Country Office, World Health Organization, Abuja, Nigeria; ^5^ Faculty of Nursing/World Health Organization Collaborating Center, Jordan University of Science and Technology, Irbid, Jordan; ^6^ Department of Psychiatry, American University of Beirut, Beirut, Lebanon; ^7^ Department of Psychiatry, United Arab Emirates University, Al Ain, United Arab Emirates; ^8^ Mental Health Services, Hamad Medical Corporation, Doha, Qatar; ^9^ Faculty of Medicine of Tunis, Tunis El Manar University, Tunis, Tunisia; ^10^ Department of Psychiatry Ibn Omrane, Razi Hospital, Manouba, Tunisia; ^11^ Department of Psychiatry, Gulf Medical University, Abu Dhabi, United Arab Emirates; ^12^ Department of Biomedical Sciences, Faculty of Medicine and Health Sciences, An-Najah National University, Nablus, West Bank, Palestine; ^13^ Department of Psychiatry, College of Medicine, Imam Abdulrahman Bin Faisal University, Dammam, Saudi Arabia; ^14^ Centre for Mental Health Research, City St. George’s, University of London, London, United Kingdom; ^15^ Department of Psychiatry and Mental Health, Hospital Clínico Universidad de Chile, Santiago, Chile

**Keywords:** psychiatric beds, residential care, forensic psychiatric care, incarceration, prison population rate, institutionalization

## Abstract

**Background:**

Psychiatric bed numbers (general, forensic, and residential) and prison populations are considered indicators of institutionalization. The present study aimed to assess trends in institutional mental health care and incarceration, measured through psychiatric bed availability and prison population rates, across the Eastern Mediterranean Region (EMR) from 1990 to 2022.

**Methods:**

We obtained retrospective data on psychiatric bed numbers and prison populations from 22 countries between 1990 and 2022. Prevalence per 100,000 population, the median prevalence and percentage changes between the first and last data points were calculated.

**Findings:**

Primary data were retrieved from 10 out of 22 countries. Data from secondary sources were used for the remaining 12 countries. In Libya and Somalia, primary data were only available for the prison populations. The median prevalence of psychiatric beds decreased from 8.8 to 5.8 per 100,000 population (-35%) between 1990 and 2022. An increase from 0.2 to 0.4 in forensic psychiatric beds was observed. The prevalence of beds in residential facilities was available from seven countries, two of which did not have beds between 1990 and 2022. The median rates increased from 0.1 to 0.2 between 1990 and 2022. The median prison population also increased from 86.9 to 108.0 per 100,000 people (+24%).

**Interpretation:**

EMR countries showed, on average, a reduction in the prevalence of psychiatric beds from low numbers, while more people were imprisoned over the past three decades. The availability of forensic psychiatric beds and residential facilities has remained limited. These findings suggest a gradual shift in the EMR from institutional psychiatric care toward increased reliance on incarceration, paralleling trends observed in other global regions like Latin America and Central Eastern Europe and Central Asia.

## 1 Introduction

The Eastern Mediterranean Region (EMR), as defined by the World Health Organization (WHO), includes a total of 21 nations and the occupied Palestinian territory (West Bank and Gaza, hereafter referred to as Palestine). The countries are Afghanistan, Bahrain, Djibouti, Egypt, the Islamic Republic of Iran (henceforth Iran), Iraq, Jordan, Kuwait, Lebanon, Libya, Morocco, Oman, Pakistan, Qatar, Saudi Arabia, Somalia, Sudan, the Syrian Arab Republic (henceforth Syria), Tunisia, the United Arab Emirates, and Yemen (1). These countries have substantial linguistic, political, historical, and sociocultural similarities but also important differences and major heterogeneity (2). Turkey was not included as it forms part of the European Region in the WHO (3).

The burden of mental illness in the EMR is greater than the global average. Gender roles in the region may contribute to a greater mental health burden among women (4). In 2020, more than 62 million people needed access to mental health care in the EMR. Part of the mental health burden is attributable to political instability, wars, terrorism, and armed conflicts in the region (5). However, mental health services are scarce; in particular, the number of psychiatric beds is low (6, 7). According to the 2020 WHO Mental Health Atlas, EMR countries had the second lowest provision of psychiatric beds in general hospitals (1.2 per 100,000 population) after the African region and the third lowest prevalence of beds in psychiatric hospitals (4.4 per 100,000) after the African and Southeast Asia regions (8). People residing in rural areas have scarce access to mental health facilities, as these facilities are usually centralized in metropolitan areas. Compared with 35% worldwide, 20% of countries reported formal collaboration with service users, their caregivers, and families (8).

Mental and substance use disorders are the principal causes of nonfatal disease burden in EMR (9). Depression and anxiety disorders account for most of this burden (10). In contrast, alcohol use (11) and suicide rates (6.4 per 100,000) (8) are the lowest worldwide. However, research in the field of substance use has recently increased after being largely neglected (12). In prison populations, mental health and substance use disorders are highly prevalent globally (13–17) and often remain underestimated, undetected, and untreated, especially in low-income and middle-income countries (LMICs) (18–20). The incidence of suicide in prison populations is typically high (21). In the EMR region, the mean prison population rate was lower (124 per 100,000) than the worldwide average (140 per 100,000) (22). However, eight of 22 countries in the region exceeded the world average (22).

In complex humanitarian crises, the prevalence of mental health problems can increase, whereas health and social systems show a limited capacity to respond. In EMR, nongovernmental organizations (NGOs) play a fundamental role in providing mental health services, especially in situations of armed conflicts (23). Psychiatric services have been gradually moving in the past decade from a few large mental hospitals to psychiatric units with inpatient and outpatient services in general hospitals. Lebanon and Iraq have conducted national studies using methods based on the World Mental Health Survey of the WHO (24, 25). However, decentralization efforts have often been limited by a lack of resources. Mental health expenditure as a percentage of the total health budget was not reported in most countries of the EMR. In the few countries for which data are available, it was below the recommended minimum to promote mental health care (26). Many countries, such as Sudan and Somalia, do not have any mental health legislation, and some do not have any mental health policy (27, 28). Furthermore, the EMR had the second lowest median rate of community-based outpatient mental health facilities (0.29 per 100,000) among all WHO regions and lower than the global median (0.55 per 100,000) (8). Thus, the provision of mental health services in the EMR is likely insufficient for meeting the service needs of the region (23).

Contemporary research has shown that lack of access to mental health services, involvement with the criminal justice system, and incarceration are linked. There is an increased risk of violent crime after the onset of several mental disorders (29, 30), and access to mental health services may reduce crime rates (31). This study aimed to assess the prevalence of psychiatric beds and the prison population across the EMR between 1990 and 2022 to allow for comparisons across jurisdictions and inform policies and service planning.

## 2 Methods

### 2.1 Establishment of the research network and participants

We formed an international network of researchers between April 2019 and July 2022. Collaborators were contacted on the basis of their scientific publications, international reports, personal networks, and snowballing. We also reached out to ministries of health and related government institutions. Researchers from 10 countries in the EMR participated in the network: Afghanistan, Iraq, Jordan, Lebanon, Pakistan, Palestine, Qatar, Tunisia, the United Arab Emirates and Yemen. The network also included a global South-South collaboration with researchers from Chile located in another emerging LMIC region. Researchers in the network communicated via email in the English language.

### 2.2 Data collection and sources

A template was used to collect national data for each year between 1990 and 2022. Data collection took place between April 2019 and July 2022. When data from primary national sources were unavailable, prison population rates were retrieved from the World Prison Brief database online (32) and psychiatric bed numbers were retrieved from the WHO Mental Health Atlas between 1990 and 2022 (33, 34).

### 2.3 Definition of psychiatric beds and prison populations

Estimates were calculated as the number of psychiatric beds and imprisoned individuals per 100,000 people based on total population counts provided by the World Bank (35). Four different indicators were assessed: 1) Psychiatric beds, which were defined as all beds in hospital settings provided for the treatment of people with mental disorders either in psychiatric hospitals or in psychiatric units of general hospitals, including beds specifically designated to treat children and adolescents with mental health problems. 2) Forensic psychiatric beds included any bed assigned for assessment or treatment in forensic psychiatry ordered by law or courts. If bed numbers for forensic psychiatric care and child and adolescent psychiatric care were stated as separate from those for general psychiatry, they were also added to the total number of psychiatric beds. In many low- and middle-income countries (LMICs), such beds are not provided separately, but general psychiatric beds are flexibly used for this purpose (36). 3) Beds in housing or residential facilities for people with mental disorders include community-based mental health care facilities that provide overnight residence, mostly providing care for patients with stable mental illnesses and patients who do not require acute medical treatment. Facilities specifically serving patients with substance use disorders or intellectual disabilities, as well as any generic facility not specifically intended for mental health treatment (e.g., rest and nursing homes for older people), were excluded. 4) Prison populations were defined as all people in full-time incarceration in prisons or jails. We excluded people on parole, probation, or serving alternative sentences during the daytime or nighttime only.

### 2.4 Statistical analysis

We calculated the percentage changes in psychiatric bed numbers and prison population rates between each country’s first and last available data points. The median values with the interquartile range (IQR) and the mean values with standard deviations between all countries were calculated at the first data points and last data points. Since the data were non-normally distributed, the medians and IQR were the primary outcomes. Changes in absolute numbers of psychiatric beds and prison populations were calculated for all countries to calculate absolute changes in the entire region since 1990. The percentage changes between the median and mean values at the first and last data points were also calculated. We compared median and mean values for *a priori* defined groups according to income level at the last data point and calculated percentage changes for the median and mean values for each income group. Finally, we compared findings in the EMR with those of countries that form part of the Organization for Economic Cooperation and Development (OECD), an international organization of 38 countries, most of which have high-income economies. Psychiatric bed numbers for OECD countries were retrieved from www.stats.oecd.org. Prison population rates of the OECD countries were retrieved from the Institute of Criminal Policy Research (www.prisonstudies.org) (32). As an exploratory approach, we calculated a prevalence ratio between incarceration rates and psychiatric beds. With respect to a therapeutic approach for people affected by addiction and dual disorders, societies may aim for low values of this ratio (i.e., approximately two as the median in the OECD). We did not involve patients or the public in the study design. Research findings will be shared with the WHO regional office and user organizations in EMR.

## 3 Results

The total population of EMR in 2022 was nearly 780 million people. Pakistan accounts for more than 30% of the total regional population. At the first data point, ten countries included in this study were lower-middle-income economies in 1990 (Djibouti, Iran, Jordan, Lebanon, Morocco, Palestine, Syria, Tunisia, and Yemen), five countries were in the low-income category (Afghanistan, Egypt, Pakistan, Somalia and Sudan), five were upper–middle–income countries (Bahrain, Iraq, Libya, Oman and Saudi Arabia), and three were high-income economies (Kuwait, Qatar and the United Arab Emirates). Fourteen countries remained in the same income group until the last data point. However, Jordan, Lebanon, and Sudan increased and then decreased again. Six countries changed to a higher income group during the observation period (Bahrain, Egypt, Iran, Oman, Pakistan, and Saudi Arabia). Syria and Yemen were the only countries that decreased from a higher income group to a lower income group between the first and last data points (**Table 1**).

**Table 1:** Eastern Mediterranean countries and their respective income group, per capita gross national income (GNI) and Gini index indicating income inequality.

Country	Population 2022	Income level 1990	Income level 2023	GNI per capita 2023^*^	Gini index	
					Year	Score
**Afghanistan**	41,128,771	LI	LI	390	ND	ND
**Bahrain**	1,472,233	UM	HI	28,280	ND	ND
**Djibouti**	1,120,849	LMI	LMI	3,450	2017	41.6
**Egypt**	110,990,103	LI	LMI	3,900	2019	31.9
**Iran**	88,550,570	LMI	UMI	4,680	2022	38.4
**Iraq**	44,496,122	UMI	UMI	5,600	2012	29.5
**Jordan**	11,285,869	LMI	LMI	4,460	2010	33.7
**Kuwait**	4,268,873	HI	HI	46,140	ND	ND
**Lebanon**	5,489,739	LMI	LMI	3,740	2011	31.8
**Libya**	6,812,341	UMI	UMI	7,570	ND	ND
**Morocco**	37,457,971	LMI	LMI	3,700	2013	39.5
**Oman**	4,576,298	UMI	HI	21,540	ND	ND
**Pakistan**	235,824,862	LI	LMI	1,500	2018	29.6
**Palestine**	4,922,749	LMI^**^	LMI	4,220	2016	33.7
**Qatar**	2,695,122	HI	HI	70,070	ND	ND
**Saudi Arabia**	36,408,820	UMI	HI	28,690	ND	ND
**Somalia**	17,597,511	LI	LI	610	2017	36.8
**Sudan**	46,874,204	LI	LI	990	2014	34.2
**Syria**	22,125,249	LMI	LI	560	2022	26.6
**Tunisia**	12,356,117	LMI	LMI	3,770	2021	33.7
**United Arab Emirates**	9,441,129	HI	HI	53,290	2018	26.0
**Yemen**	33,696,614	LMI	LI	820	2014	36.7

LI, Low-Income; LMI, Lower Middle-Income; UMI, Upper Middle-Income; HI, High Income; ND no data.

^*^Atlas method (current US$); Yemen 2018: GNI only available until 2018; Kuwait until 2019; Bahrain, Saudi Arabia, Syria and United Arab Emirates until 2020. ^**^Data from 1994 onward.

All data retrieved from World Bank webpage, 2024 (37).

Primary data on the prevalence of psychiatric beds and prison populations were retrieved from 10 out of 22 countries in the EMR (Afghanistan, Iraq, Jordan, Lebanon, Pakistan, Palestine, Qatar, Tunisia, the United Arab Emirates, and Yemen). For each of the remaining twelve countries, data were retrieved from secondary sources. No reliable data regarding the prison population were retrieved for Somalia (**Table 2**). Primary data on residential facilities were available in five countries, and data on specialized forensic psychiatric beds were available in eight countries.

**Table 2:** Prevalence of psychiatric beds, specialized forensic psychiatric beds, places in residential facilities for individuals with mental health problems, and prison populations in 22 countries in the Eastern Mediterranean Region between 1990 and 2022.

Country	Psychiatric beds per 100,000 population				Specialized forensic psychiatric beds per 100,000 population				Residential facilities per 100,000 population				Prison population per 100,000 population			
	Period of observation	First data point	Last data point	Percentage change	Period of observation	Rate at first point	Rate at last point	Percentage change	Period of observation	Rate at first point	Rate at last point	Percentage change	Period of observation	Rate at first point	Rate at last point	Percentage change
Afghanistan	1990-2022	0.6	0.4	-20	1990-2024	0.0	0.0	0	1990-2020	0.0	2.1	NA	2004-2024	21	46	117
Bahrain	1996-2020	31.8	16.3	-49	NA	ND	ND	ND	1990-2020	0.0	0.0	0	1993-2017	53	239	350
Djibouti	2001-2020	7.0	4.1	-41	NA	ND	ND	ND	1990-2020	0.0	0.0	0	1994-2018	104	63	-40
Egypt	1990-2017	13.1	7.2	-45	2006-2016	0.9	0.6	-33	1990-2020	0.0	0.0	0	1992-2022	56	110	95
Iran	2001-2020	12.4	12.3	-1	1990-2022	0.0	0.0	0	2006-2020	5.1	26.3	420	1993-2020	170	217	27
Iraq	1990-2020	8.8	3.5	-61	1990-2021	1.4	0.6	-59	1990-2020	0.0	0.0	0	2004-2021	27	179	573
Jordan	1996-2020	11.8	4.7	-61	2005-2020	0.7	0.8	9	1990-2020	0.0	0.0	0	1993-2022	92	170	84
Kuwait	1998-2020	47.3	20.5	-57	1990-2020	0.0	0.9	NA	1990-2020	0.0	0.0	0	1997-2023	98	105	7
Lebanon	1996-2020	44.6	24.5	-45	1990-2020	0.0	0.5	NA	1990-2020	0.0	1.6	NA	1993-2023	66	169	156
Libya	1990-2016	37.8	31.8	-16	NA	ND	ND	ND	NA	ND	ND	ND	1998-2023	136	280	107
Morocco	1998-2020	6.5	5.8	-10	1990-2020	0.0	0.0	0	1990-2020	0.0	0.0	0	1990-2023	134	276	105
Oman	1990-2021	4.9	6.2	27	1990-2021	0.0	0.4	NA	1990-2021	0.0	0.0	0	1998-2015	84	47	-44
Pakistan	1997-2020	1.8	5.7	212	NA	ND	ND	ND	NA	ND	ND	ND	1993-2022	55	37	-32
Palestine	1990-2022	13.4	4.4	-67	1990-2022	0.3	0.2	-22	1990-2022	0.0	0.0	0	2000-2018	83	142	70
Qatar	1990-2022	0.0	4.5	NA	1990-2022	0.0	0.6	NA	1990-2022	0.0	1.2	NA	1993-2019	105	58	-45
Saudi Arabia	1996-2022	10.3	13.4	30	2013-2020	0.2	0.6	244	2011-2020	0.8	1.5	93	1998-2017	113	199	76
Somalia	2001-2020	0.3	1.3	402	NA	ND	ND	ND	1990-2020	0.0	1.0	NA	NA	ND	ND	ND
Sudan	1998-2017	0.1	0.7	591	2006-2012	0.6	0.6	-13	2006-2012	1.8	2.1	20	2002-2017	46	52	11
Syria	2001-2020	8.0	4.3	-46	NA	ND	ND	ND	NA	ND	ND	ND	1997-2004	92	59	-36
Tunisia	1990-2020	9.6	8.2	-15	1990-2020	0.6	0.4	-35	1990-2020	0.0	1.4	NA	1996-2021	246	192	-22
United Arab Emirates	1990-2022	15.8	5.6	-65	1990-2022	0.5	0.3	-42	1990-2022	0.0	0.0	0	1998-2014	205	111	-46
Yemen	1990-2022	4.9	3.3	-33	1990-2022	0.4	0.7	83	NA	ND	ND	ND	1990-2019	33	45	38
MEDIAN^*^		9.2	5.7	-38		0.2	0.5	161		0.0	0.0	NA		92	111	21
IQR		8.4	7.1	-16		0.6	0.3	-52		0.0	1.5	NA		58	133	129
MEAN^*^		13.2	8.6	-35		0.3	0.4	26		0.4	2.1	389		96	133	38
SD		14.1	8.1	-43		0.4	0.3	-36		1.2	6.1	393		58	80	39
Absolute numbers (thousands)		32.4	50.1	54		1.1	2.2	25		4.3	25.5	481		330	792	140
Total population (millions)		436	741	70		287	462	60		272	419	54		430	730	70
Number of countries		22	22			16	16			18	18			21	21	

NA, not applicable; ND, no data; IQR, interquartile range; SD, standard deviation.

* Median and mean changes are based on variable observation periods due to data availability.

### 3.1 Psychiatric beds

**Figure 1:**
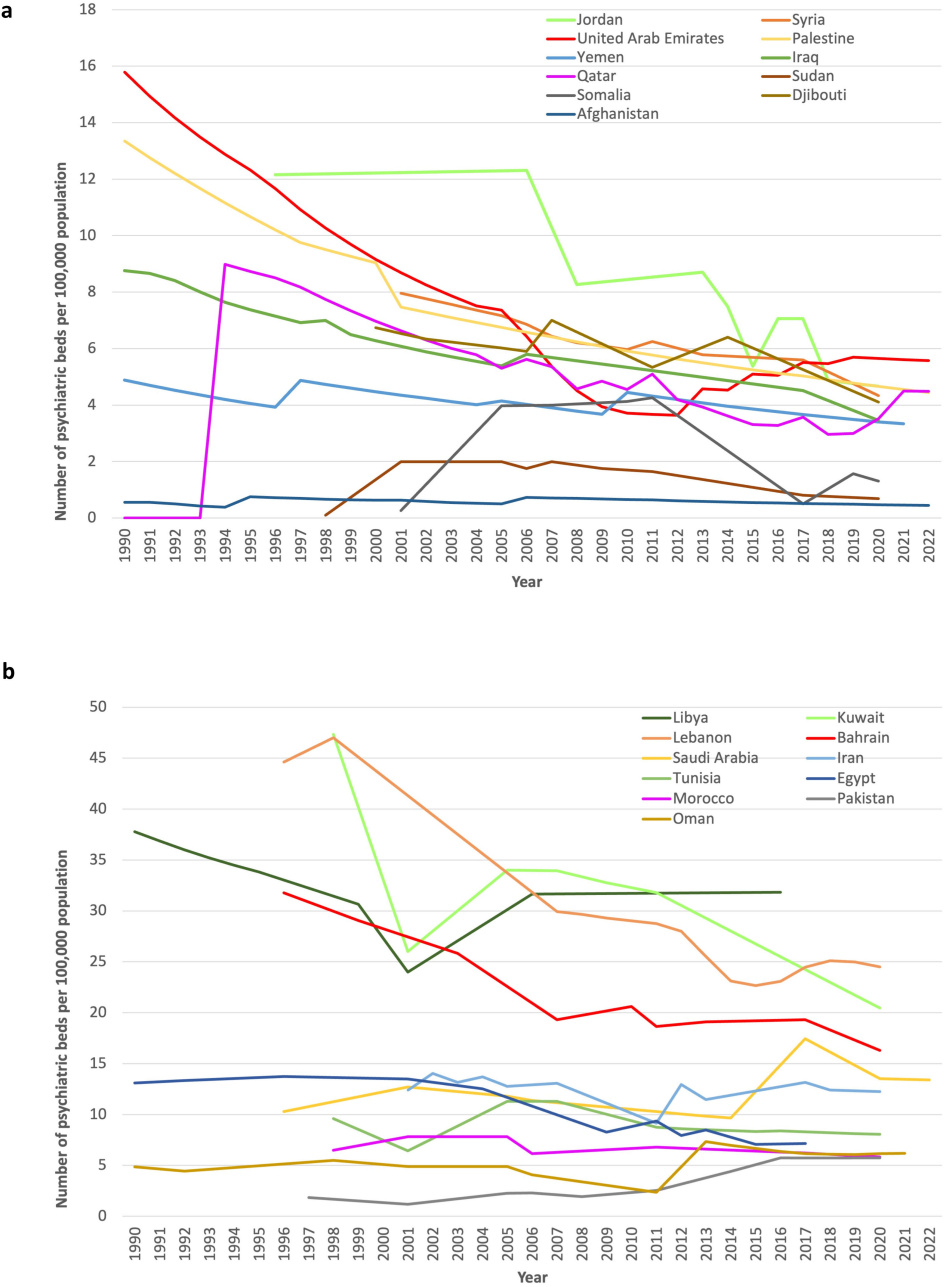
Prevalence of psychiatric beds per 100,000 people in the EMR region (1990 to 2022). For better readability of the graphs, the countries were divided into two groups at the median value of 5.7 psychiatric beds per 100,000 people at the last data point. **(a)** Countries with fewer than 5.7 psychiatric beds per 100,000 people at the last data point. **(b)** Countries with more than 5.7 psychiatric beds per 100,000 people at the last data point.

The median prevalence of psychiatric beds decreased from 9.2 to 5.7 per 100,000 population between 1990 and 2022 (-3.5 per 100,000 population; -38%), ranging from the highest percentage increase in Sudan (+591%; +0.6 beds per 100,000) to the strongest percentage decrease in the United Arab Emirates (-65%, -10.2 beds per 100,000) (**Table 2**). Most countries (16 out of 22) presented a decrease in psychiatric bed prevalence between the first and last data points during the observation period (**Figures 1a, b**). Four out of six countries exhibited increases in bed capacity (Pakistan, Qatar, Somalia, and Sudan), but the numbers were still low at the end of the observation period (below the global median). Afghanistan, Somalia, and Sudan had the lowest prevalence of psychiatric beds, all of which were less than 1.4 per 100,000 people at the last data point. Libya (31.8), Lebanon (24.5) and Kuwait (20.5) had the highest prevalence of psychiatric beds in the region, albeit all of them tended toward lower bed capacities.

In the entire region, the total number of psychiatric beds increased from 32,439 at the first data point to 50,063 at the last data point (+54%, while the growth of the total population in the EMR was +70%).

### 3.2 Prison population

**Figure 2:**
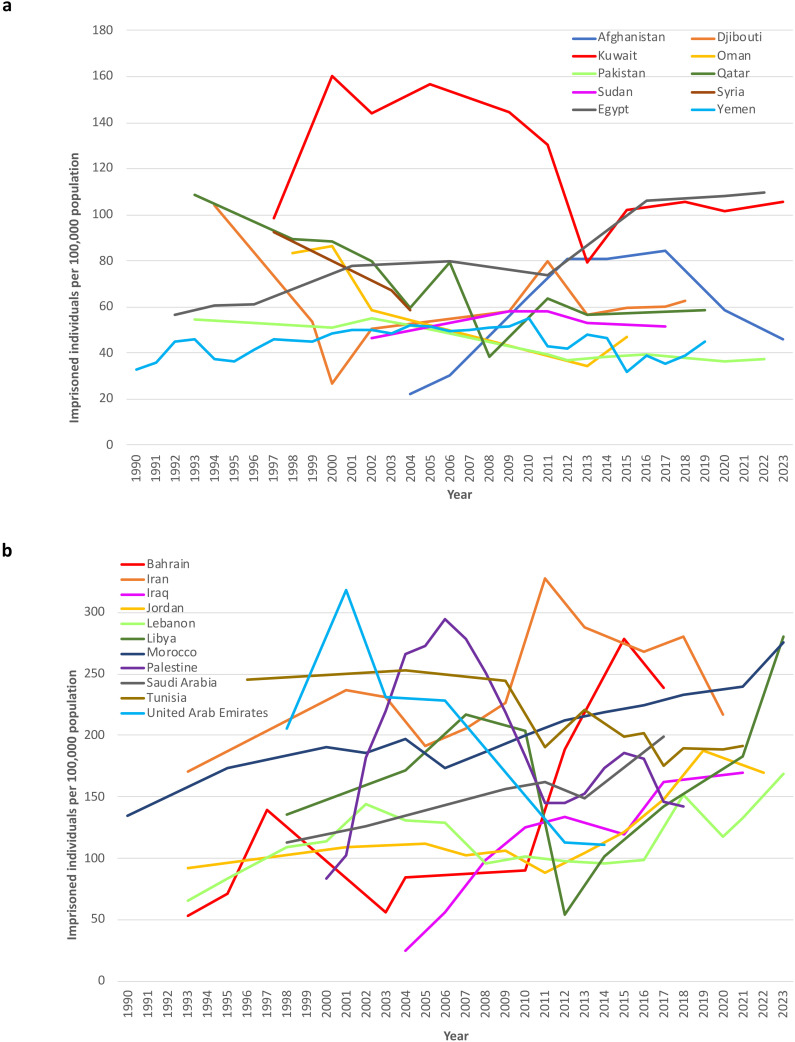
Imprisoned people per 100,000 people in the EMR region (1990 to 2022). For better readability of the graphs, the countries were divided into two groups at the median value of 111 imprisoned people per 100,000 people at the last data point. **(a)** Countries with fewer than 111 imprisoned individuals per 100,000 people at the last data point. **(b)** Countries with more than 111 imprisoned people per 100,000 people at the last data point.

The median prevalence of imprisonment increased from 92 at the first data point to 111 per 100,000 population at the last data point between 1990 and 2022 (median percentage change +21%; +19.2 per 100,000 population) (**Figures 2a, b**). The prevalence of imprisonment increased in 14 countries and decreased in seven countries, ranging from the highest increase of 573% in Iraq (+152.4 per 100,000 population) to the strongest decrease of approximately -45% in Oman (-37 imprisoned individuals per 100,000 population), Qatar (-47 imprisoned individuals per 100,000 population), and the United Arab Emirates (-94 imprisoned individuals per 100,000 population).

The estimated total number of imprisoned individuals reported in the region was 330,245 at the first data point and 791,717 at the last data point (+140%). No data were available for Somalia over the observation period.

### 3.3 Forensic psychiatric beds

**Figure 3:**
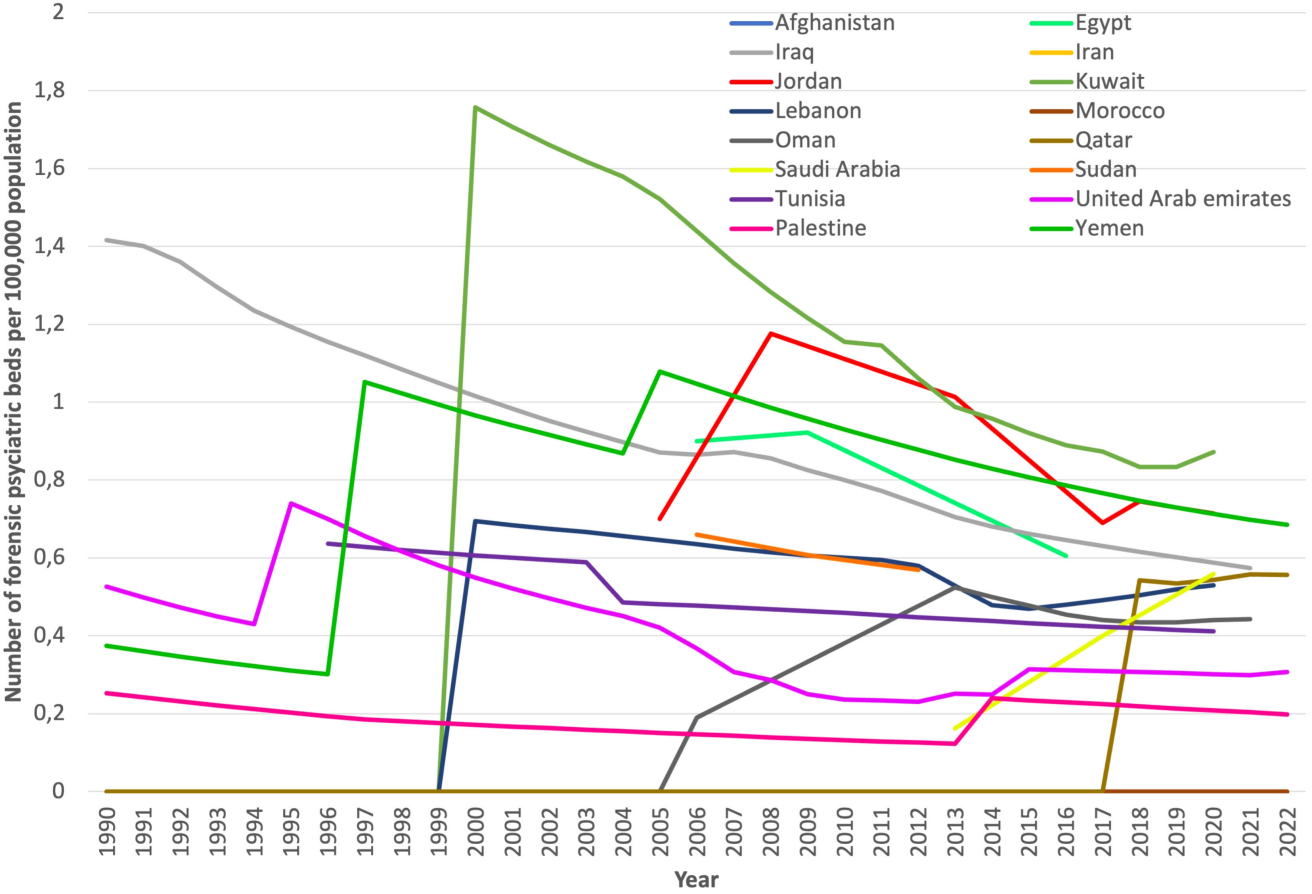
Prevalence of forensic psychiatric beds per 100,000 people in the EMR region (1990-2022).

We included data from 16 countries on this indicator, mostly from secondary sources. Afghanistan and Morocco reported not having had any forensic beds between 1990 and 2022 (**Table 2**). In the remaining fourteen countries, the forensic psychiatric bed prevalence ranged from 0.2 to 0.9 per 100,000 population at the last data point (**Figure 3**). The median prevalence of forensic psychiatric beds increased from 0.21 to 0.55 per 100,000 population between the first and last data points (median percentage change +167%; +0.35 per 100,000 population). Iran, Kuwait, Lebanon, Oman and Qatar introduced forensic psychiatric beds during the observation period. Jordan (+0.1 beds per 100,000), Saudi Arabia (+0.4 beds per 100,000) and Yemen (+0.3 beds per 100,000) reported an increase in forensic psychiatric beds. However, Egypt, Iraq, Palestine, Sudan, Tunisia, and the United Arab Emirates reported a decrease in the prevalence of forensic beds. The total number of forensic beds in the region was 1,739 at the first data point and 2,175 at the last data point (+25%). In Pakistan, some hospitals and prisons are equipped with forensic psychiatric beds, although there is inconsistent information on the number of available beds (38).

### 3.4 Places in residential facilities

Prevalence data for beds in residential facilities were obtained for 18 countries, most of which were from secondary sources. Ten countries (Bahrain, Djibouti, Egypt, Iraq, Jordan, Kuwait, Morocco, Oman, Palestine and the United Arab Emirates) reported the absence of residential facilities between 1990 and 2022 (**Table 2**). Afghanistan (+2.1 per 100,000), Lebanon (+1.6 per 100,000), Qatar (+1.0 per 100,000), Somalia (+1.0 per 100,000) and Tunisia (+1.5 per 100,000) started implementing residential facilities during the observation period. Iran, Saudi Arabia (+0.8 per 100,000) and Sudan (+0.3 per 100,000) increased the capacity of places in residential facilities.

Data on residential facilities were excluded from Libya, Pakistan, and Yemen. These facilities may provide services to people with intellectual disabilities, for detoxification, or to the homeless. Therefore, these numbers may not be comparable to those reported in other countries.

The estimated total number of reported beds in residential facilities for people with mental disorders at the first data point was 4,390, and it was 25,495 at the last data point for the entire region (+481%). Close to 90% of the capacities in the region were located in Iran.

### 3.5 Assessments by income group

The assessment by income groups revealed an increase in median psychiatric bed numbers between the first and last data points among low-income countries, albeit at very low levels (lower than 1.3 beds per 100,000 people). Middle- and high-income countries tended to have lower psychiatric bed capacities (**Table 3**).

**Table 3:** Prevalence of psychiatric inpatient beds and prison populations by income group in Eastern Mediterranean countries between first and last data points.

Income group (2021)		Psychiatric beds per 100,000 population			Prison population per 100,000 population		
		At first point	At last point	% change	At first point	At last point	% change
Low-Income (Afghanistan, Somalia,^*^ Sudan, Syria, and Yemen)	Median	0.6	1.3	133	40	49	24
	Number of countries	5	5		4	4	
Middle-Income (Djibouti, Egypt, Iran, Iraq, Jordan, Lebanon, Libya, Morocco, Pakistan, Palestine and Tunisia)	Median	11.8	5.8	-51	92	170	84
	Number of countries	11	11		11	11	
High-Income (Bahrain, Kuwait, Oman, Qatar, Saudi Arabia, and United Arab Emirates)	Median	13.0	9.8	-25	102	108	6
	Number of countries	6	6		6	6	

*Somalia excluded from prison population rates.

First and last data point statistics may not rigorously express the evolution between 1990 and 2022 due to incompleteness of data over the timespan.

Concerning the prison population, a strong increase was observed in middle-income countries (almost doubling the incarceration rates). A substantial increase in the median incarceration rates from low initial levels was observed in low-income countries. High-income countries had almost unchanged median incarceration rates during the observation period.

### 3.6 Ratio between the prison population and psychiatric beds

Iraq, Morocco, and Sudan presented the highest ratios, with more than 40 imprisoned individuals per psychiatric bed at the last data point. Conversely, Kuwait, Lebanon, Oman, and Pakistan had the lowest ratios, all below 8 imprisoned people per psychiatric bed or lower, at the last data point.

**Figure 4:**
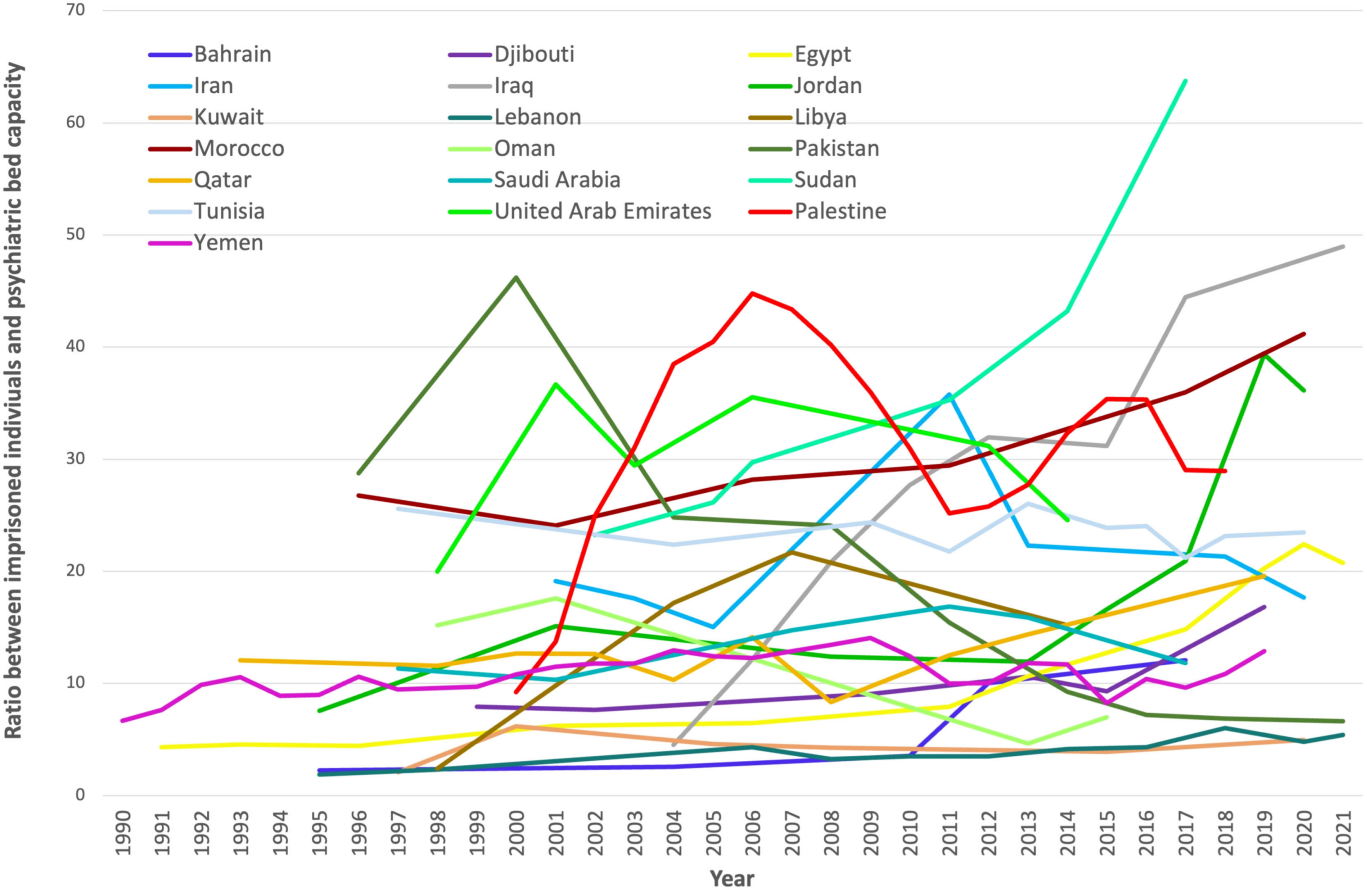
The prevalence ratio between imprisoned individuals and psychiatric beds in Eastern Mediterranean countries (1990 to 2021).

With respect to the evolution of this indicator, 15 out of 19 countries experienced increases, whereas four experienced decreases in this ratio. Bahrain, Iraq, and Libya showed the strongest increases, whereas a strong decrease was observed only in Oman and Pakistan (**Figure 4**). Iran and Tunisia also experienced small decreases in this indicator.

Three countries were excluded from this analysis. Afghanistan was an outlier, with more than 150 imprisoned individuals per psychiatric bed. Only one data point for this ratio was available for Syria, and no prison population rates were available to calculate this ratio in Somalia.

### 3.7 Comparison with organization for economic co-operation and development countries

**Figure 5:**
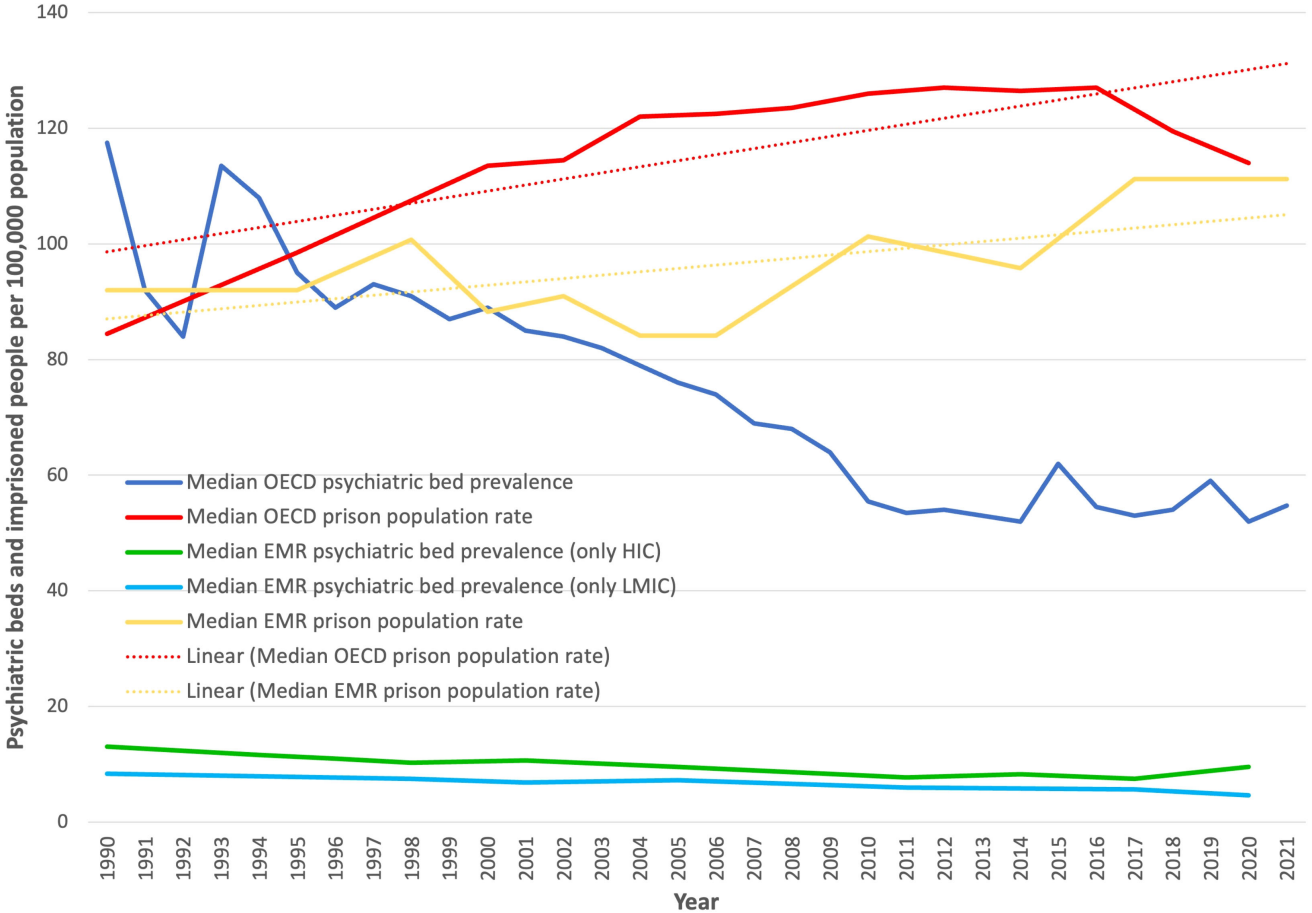
Median psychiatric bed and prison population prevalence in EMR countries compared with that in OECD countries (1990 to 2021).

The median number of psychiatric beds per 100,000 people decreased in OECD countries and in the EMR between 1990 and 2020. However, in 2020, the median prevalence of psychiatric beds in the EMR was approximately ten times lower than that in OECD countries (5.7 vs 54.8 per 100,000 population) (**Figure 5**). The psychiatric bed prevalence in HIC of the EMR was still considerably lower than the median OECD bed prevalence.

On the other hand, the mean prison population in the EMR showed an increasing trend, which was also observed in OECD countries. The median prison population at the last data point (2020, based on countries with data available for 2019, 2020, or 2021) in the EMR was similar to the median prison population in OECD countries (114 per 100,000 people) (**Figure 5**).

## 4 Discussion

### 4.1 Main findings

The prevalence of psychiatric beds decreased in fifteen countries and increased in seven countries of the EMR. In the HIC of the region, the bed prevalence remained much lower than that of the OECD. Prison population rates increased in fourteen out of 22 countries to levels close to the OECD average; thus, an increase in the ratio between the prison population and psychiatric beds was observed in the EMR. Data are scarce in many countries, especially regarding forensic beds and residential places.

### 4.2 Interpretation

Worldwide, need estimates of psychiatric beds are increasingly higher than the actual number of provided beds (39). The number of psychiatric beds was alarmingly low compared with that in OECD countries, and compared with recommendations based on international expert consensus (7). Nevertheless, the median psychiatric bed prevalence was higher than that in sub-Saharan Africa (40), where budgets for public health provision are typically lower. However, the bed prevalence was still lower than that in other regions with LMICs, such as Latin America (41), Central Eastern Europe and Central Asia (3). Among the twenty-two EMR countries, only Kuwait and Lebanon provided more than the global median in 2020 (14.5 beds per 100,000 population) (8) at the last data point. Mental health services are still predominantly based in psychiatric hospitals in the region, which should undergo reforms to achieve better integration in general hospitals, with community mental health services and decentralization (42).

Additional dynamic factors are relevant for understanding the mechanisms underlying the use and need for psychiatric beds. For example, long-stay inpatient treatments are more common than in the rest of the world (17% vs 9% of patients who have stayed for more than one year in inpatient treatments) (42). This could indicate that a proportion of the patients in inpatient hospital units may be placed in community residential services if these facilities are adequately developed. On the other hand, a significant number of people with mental illness who need acute treatment may find it harder to get a bed due to lower admission turnover. When people with decompensated psychosis and/or substance use disorder in the context of poorly resourced community treatment settings and unavailability of acute care beds show violent conduct, the risk of imprisonment is substantial. Low-income countries in the EMR have lower psychiatric bed prevalence and incarceration rates than middle- and high-income countries within the region. It has been hypothesized that governments from LMICs are usually not able to pay for any type of institutional care (43). LMICs usually have less mental health infrastructure availability; thus, the absence of an increase or reduction in the number of psychiatric beds is unlikely to be compensated with residential and community services. High-income countries in the region (Gulf Cooperation Council member states) attract large numbers of migrant workers. The prevalence of psychiatric morbidity among migrants is greater than that among nationals, which needs consideration for service development (44).

The low prevalence of beds throughout the region may be due not only to socioeconomic factors but also to other cultural variables, such as barriers to mental health. A lack of mental health awareness and literacy (45), as well as stigma among patients, families, and care providers, are observed in EMR countries (46). Feelings of shame about the mental health condition and treatment needs can also constitute a barrier to service use and reduce the demand for beds. Different coping styles among patients and families with mental illness can also become a barrier to seeking mental health treatment (47). Additionally, cultural gender norms can adversely affect women’s access to mental health treatment (48). Sociopolitical instability in the region puts additional stress on the functioning of mental health institutions. The presence of beds in mental health institutions in this context does not imply that they can be properly operated, thus, the shortage could be underestimated.

The relationship between incarceration and psychiatric infrastructure has long been described as particularly relevant for people with addictions and dual disorders (49). To avoid disproportionate developments between prison and mental health infrastructure with devastating social, economic, and health consequences, the ratio between incarceration rates and psychiatric bed prevalence may guide government planning. This ratio is typically close to one in Western Europe (50) and approximately two in the OECD.

When comparing EMR with OECD countries, we observed a faster increase in median incarceration rates and a less pronounced decrease in median psychiatric beds. At the end of the observation period, incarceration rates in EMRs were higher than those in OECD countries, while OECD countries had a ten times higher psychiatric bed prevalence. The increasing ratio of the prison population to psychiatric beds may mean that people with mental illness and disruptive behaviors may be more likely to be criminalized in the correctional system rather than treated during psychiatric hospitalization. This could indicate an imbalance in service development.

### 4.3 Strengths and limitations

This is the first study to assess changes in indicators of institutionalization in twenty-two Eastern Mediterranean countries over three decades and to assess the balance of development between different types of institutions, such as prison populations and psychiatric beds. The study includes views from researchers based in 10 EMR countries and a global South-South collaboration.

The lack of primary data sources from twelve of the twenty-two countries in the region was a limitation. In six countries, we did not obtain any data on forensic psychiatric bed availability. In four countries, no data on residential bed capacities were available and in Somalia, we did not obtain any information on the incarceration rates. A limited period of observation due to missing data points was common for several countries and indicators, especially forensic beds and residential facilities. Therefore, caution is warranted when comparing percentage changes between countries. Even though it is a strength of this study to include collaboration and views from researchers based in 10 EMR countries, future research may achieve wider collaborations in the region, spanning more countries, thus reducing the number of missing data points. Variable definitions of residential facilities also limit comparisons between countries. Nevertheless, this study is a starting point for service comparisons across jurisdictions in the region and beyond.

### 4.4 Implications

In conclusion, psychiatric beds were removed or added at a slower rate than population sizes increased in most countries in the EMR, exacerbating the scarcity and likely critical shortage in most countries. In contrast, incarceration rates increased on average, possibly reflecting a more penalizing treatment of people with addiction and deviant behaviors. EMR countries should strengthen community psychiatric care, including outreach services for people with severe mental illness and rehabilitation services for people with substance use disorders, to prevent institutionalization. Those services should reach people with criminal justice involvement. The development of specialized care options for people with mental health conditions in prisons also needs consideration.

The scarcity of forensic psychiatric bed capacities should receive special attention for service development, since legally incompetent individuals with severe mental illness may not be diverted from imprisonment with consequences for the safety and human rights of those vulnerable people. Future developments in psychiatric service infrastructure should consider the sector of inpatient treatments to complement community and outpatient care. Development in psychiatric infrastructure lags relative to the expansion in size observed in correctional systems. A novel index reflecting the ratio of the two indicators could be used to set new development goals.

## Data Availability

The original contributions presented in the study are included in the article/**Supplementary Material**. Further inquiries can be directed to the corresponding author.
