# Supplementary material for: Changes in the provision of psychiatric beds and incarceration in the Eastern Mediterranean Region from 1990 to 2022
Source: Front Psychiatry. 2025 Jul 16;16:1539662. doi: 10.3389/fpsyt.2025.1539662 (PMC12307341; doi:10.3389/fpsyt.2025.1539662)
Supplement: Supplementary file 1 [file Table1.docx]

**Table S1. Data sources for bed availability and prison population in countries from EMRO.**

|  | **WHO Mental**  **Health Atlas^±^** | **Publications by**  **Okasha et al.^*^** | **World Prison Brief** | **Additional sources on bed availability or prison population** |
| --- | --- | --- | --- | --- |
| **Afghanistan** | X |  | X | - Direct data obtained by Akbar Paiman at the Mental Health Department of the Ministry of Public Health of Afghanistan. - WHO-AIMS Report on Mental Health System in Afghanistan, WHO and Ministry of Public Health, Kabul, Afghanistan, 2006. |
| **Bahrain** | X | X | X | - Al-Haddad MK, Al-Offi A. Psychiatric services in Bahrain: past, present and future. Int Psychiatry. 2009 Jan 1;6(1):14-16. - WHO-AIMS Report on Mental Health System in Kingdom of Bahrain, WHO and Ministry of Health, Manama, Kingdom of Bahrain, 2010. |
| **Djibouti** | X | X | X | - Le rapport de l’OMS-IESM sur le système de Santé Mentale a Djibouti, l’OMS et le Ministère de la Santé, (Djibouti ville, Djibouti), 2006. |
| **Egypt** | X | X | X | - Okasha, A. (1991) "Mental Health Services in Egypt," The Journal of Sociology & Social Welfare: Vol. 18: Iss. 2, Article 6. - Okasha A. Psychiatry in Egypt. *Psychiatric Bulletin*. 1993;17(9):548-551. - Saeed, Sally Ibrahim Noby. AIMS OF EGYPT Assessment of Governmental Mental Health System Egypt (2016-2017). New University of Lisbon, Faculty of Medical Sciences; World Health Organization, Department of Mental Health and Substance Deficiency Faculdade de Ciências Médicas, 2018. URL: <http://hdl.handle.net/10362/40174> - WHO-AIMS Report on Mental Health System in (Egypt), WHO and Ministry of Health, Cairo, Egypt, 2006. - Elnemais Fawzy, Michael. (2017). Mental health care in Egypt: Review of current state, policy, and needs. International Journal of Mental Health. 46. 1-7. |
| **Iran** | X |  | X | - WHO-AIMS Report on Mental Health System in The Islamic Republic of Iran, WHO and Ministry of Health and Medical education, Tehran, the Islamic Republic of Iran, 2006. - Damari B, Alikhani S, Riazi-Isfahani S, Hajebi A. Transition of Mental Health to a More Responsible Service in Iran. Iran J Psychiatry. 2017 Jan;12(1):36-41. - Sadeghi M, Mirsepassi G. Psychiatry in Iran. Int Psychiatry. 2005 Oct 1;2(10):10-12. - Noorbala AA, Bagheri Yazdi SA, Faghihzadeh S, Kamali K, Faghihzadeh E, Hajebi A, Akhondzadeh S, Esalatmanesh S, Bagheri Yazdi HS, Abbasinejad M, Asadi A. Trends of mental health status in Iranian population aged 15 and above between 1999 and 2015. Arch Iran Med. 2017; 20(11 Suppl. 1): S2 – S6. - Beis P, Graf M, Hachtel H. Impact of Legal Traditions on Forensic Mental Health Treatment Worldwide. Front Psychiatry. 2022 Apr 25;13:876619. |
| **Iraq** | X | X | X | - Humaidi, Naamah. (2006). Resettlement Prospects for Inpatients at Al Rashad Mental Hospital. Journal of Muslim Mental Health. 1. 177-183. - WHO-AIMS Report on Mental Health System in Iraq, WHO and Ministry of Health, Baghdad, Iraq, 2006. - Mahmood, Muhamed. (2021). Violence Among Schizophrenic Pateints In AlRashad Training Hospital. Kirkuk Journal of Medical Sciences. 9. 218-250. - Sadik A. A snapshot of Iraqi psychiatry. BJPsych Int. 2021 Feb;18(1):9-11. Erratum in: BJPsych Int. 2022 Aug;19(3):80. |
| **Jordan** | X | X | X | - Hijiawi B, Elzein Elmousaad H, Marini A, Funk M, Skeen S, Al Ward N, Saeed K. Ayoub Z. WHO Profile on mental health in development (WHO proMIND): Hashemite Kingdom of Jordan. Geneva, World Health Organization, 2013. - WHO-AIMS Report on Mental Health System in Jordan, WHO and Ministry of Health, Amman, Jordan, 2011. - International Medical Corps - Understanding the Mental Health and Psychosocial Needs, and Service Utilization of Syrian Refugees and Jordanian Nationals: A Qualitative & Quantitative Analysis in the Kingdom of Jordan (2017). |
| **Kuwait** | X | X | X | - Zahid MA, Al-Zayed A. Psychiatry in Kuwait. Int Psychiatry. 2009 Apr 1;6(2):34-36. - S. Eid, P02-55 - Forensic psychiatric services in Kuwait and the need for mental health legislation, European Psychiatry, Volume 25, Supplement 1, 2010, Page 675, ISSN 0924-9338. - Alhumoud A, Zahid M, Ibrahim S, Syed T, Naguy A. Forensic psychiatry in Kuwait - characterization of forensic psychiatry patients evaluated over year duration in the only available forensic psychiatry unit. Int J Law Psychiatry. 2018 Sep-Oct;60:12-16. |
| **Lebanon** | X | X | X | - WHO-AIMS Report on Mental Health System in Lebanon, WHO and Ministry of Health, (Beirut, Lebanon, 2010). - Ghossoub E, Felthous AR. Mental illness and the Lebanese criminal justice system: Practices and challenges. Int J Law Psychiatry. 2020 Jul-Aug;71:101604. |
| **Libya** | X | X | X | - Abuazza A. The Arab Spring movement: a catalyst for reform at the psychiatric hospital in Tripoli, Libya. Int Psychiatry. 2013 Aug 1;10(3):56-58. - Rhouma AH, Husain N, Gire N, Chaudhry IB. Mental health services in Libya. BJPsych Int. 2016 Aug 1;13(3):70-71. - El-Badri SM. Psychiatry in Libya: eastern region. *Psychiatric Bulletin*. 1995;19(1):48-49. |
| **Morocco** | X | X | X | - Hamaoui YE, Moussaoui D, Okasha T. Forensic psychiatry in north Africa. Curr Opin Psychiatry. 2009 Sep;22(5):507-10. - WHO-AIMS Report on Mental Health System in Morocco, WHO and Ministry of Health, Rabat, Morocco, 2006. |
| **Oman** | X | X | X | - Ministry of Health. *Al Masarra Hospital*. Ministry of Health, Sultanate of Oman, 2023 (<https://www.moh.gov.om/en/web/almassara-hospital>) - Al-Sinawi H, Mirza H. Psychiatry in the sultanate of Oman. BJPsych Int. 2023 Nov;20(4):81-84. - WHO-AIMS Report on Mental Health System in Oman, WHO and Ministry of Health, Muscat, Oman, 2008. - Burjorjee R, Al-Adawi S. The Sultanate of Oman: an experiment in community care. *Psychiatric Bulletin*. 1992;16(10):646-648. |
| **Pakistan** | X |  | X | - Routledge Bhugra, D & Tse, Samson & Ng, R.M.K. & Takei, Nori. (2015). Routledge Handbook of Psychiatry in Asia. - Yousaf, F. (1997). Psychiatry in Pakistan. International Journal of Psychiatry 43(4), 298-302. - WHO-AIMS Report on Mental Health System in Pakistan, WHO and Ministry of Health, Islamabad, Pakistan, 2009. |
| **Palestine** |  |  |  | - Data retrieved from Palestinian Ministry of Health (by Mohammad Marie) - Palestinian Central Bureau of Statistics (PCBS), 2024. (available online at <https://www.pcbs.gov.ps/site/lang__en/1288/default.aspx?lang=en>) |
| **Qatar** | X |  | X | - Ghuloum S, Ibrahim MA. Psychiatry in Qatar. Int Psychiatry. 2006 Oct 1;3(4):16-18. - El-Islam, M.. (1995). Psychiatry in Qatar. Psychiatric Bulletin. 19. 779-781. - Ouanes S, Becetti I, Ghuloum S, Hammoudeh S, Shehata M, et al. (2020) Patterns of prescription of antipsychotics in Qatar. PLOS ONE 15(11): e0241986. - Alabdulla M, Reagu SM, Tulley I. Establishing comprehensive forensic mental health services in Qatar. Asian J Psychiatr. 2021 Mar;57:102590. - Huma Iram (2019). Psychiatric Patients Boarding at the Emergency Department: Length of Stay and Consequences. Journal of Medical & Clinical Research 4(4):1-6. - Sharkey T. Mental health strategy and impact evaluation in Qatar. *BJPsych International*. 2017;14(1):18-21. - Mohammed M, Makki I, Ghuloum S. Psychiatry in Qatar. BJPsych Int. 2023 Nov;20(4):79-81. |
| **Saudi Arabia** | X | X | X | - Al-Habeeb AA, Qureshi NA. Mental and Social Health Atlas I in Saudi Arabia: 2007-08. East Mediterr Health J. 2010 May;16(5):570-7. - Ministry of Health – Statistical Yearbook 2022 (available online at <https://www.moh.gov.sa/en/Ministry/Statistics/book/Pages/default.aspx>) - Koenig, H. , Al Zaben, F. , Sehlo, M. , Khalifa, D. , Al Ahwal, M. , Qureshi, N. and Al-Habeeb, A. (2014) Mental Health Care in Saudi Arabia: Past, Present and Future. *Open Journal of Psychiatry*, **4**, 113-130. - Qureshi NA, Al-Habeeb AA, Koenig HG. Mental health system in Saudi Arabia: an overview. Neuropsychiatr Dis Treat. 2013;9:1121-35. Epub 2013 Aug 8. |
| **Somalia** | X |  |  | - WHO-AIMS Report on Mental Health System in Somaliland region of Somalia, WHO, GAVO, and Ministry of Health, Hargeisa, Somaliland region of Somalia, 2009. - Syed Sheriff RJ, Reggi M, Mohamed A, Haibe F, Whitwell S, Jenkins R. Mental health in Somalia. Int Psychiatry. 2011 Nov 1;8(4):89-91. |
| **Sudan** | X | X | X | - Osman AHM, Bakhiet A, Elmusharaf S, Omer A, Abdelrahman A. Sudan’s mental health service: challenges and future horizons. *BJPsych International*. 2020;17(1):17-19. - WHO-AIMS Report on Mental Health System in Sudan, WHO and Ministry of Health, Khartoum, Sudan 2009. - Waging Peace – Mental Health in Sudan – The Psychiatric & Psychological Infraestructure (available online at [www.wagingpeace.info/wp-content/uploads/pdfs/Mental_health_in_Sudan_-_the_psychiatric_infrastructure.pdf](http://www.wagingpeace.info/wp-content/uploads/pdfs/Mental_health_in_Sudan_-_the_psychiatric_infrastructure.pdf)) |
| **Syria** | X |  | X | - Abou-Saleh M, Mobayed M. Mental health in Syria. Int Psychiatry. 2013 Aug 1;10(3):58-60. - Assalman I, Alkhalil M, Curtice M. Mental health in the Syrian Arab Republic. Int Psychiatry. 2008 Jul 1;5(3):64-66. |
| **Tunisia** | X | X | X | - WHO-AIMS Report on Mental Health System in Tunisia, WHO and Ministry of Health, Tunis, Tunisia, 2008. |
| **United Arab Emirates** | X |  | X | - Eapen V, El-Rufaie O. United Arab Emirates (UAE). Int Psychiatry. 2008 Apr 1;5(2):38-40. |
| **Yemen** | X |  | X | - Qasem Saleh MAB, Makki AM. Mental health in Yemen: obstacles and challenges. Int Psychiatry. 2008 Oct 1;5(4):90-92. - WHO Yemen. National Mental Health Strategy in Yemen, 2022-2026. October, 2022. Available online at: <https://www.mhinnovation.net/resources/national-mental-health-strategy-yemen-2022-2026>   Data gathered by Maan Saleh based on the following sources:   - Ministry of Health (2007): Report of the National Mental Health Program, unpublished - Ministry of Interior affair (2010-15) Annual Reports Ministry of Interior / Department of Rehabilitation and Correction (annual information for central correctional facilities in the governorates of the Republic of Yemen. Sana'a) - Ministry of Social Affairs (2000-2015): Annual reports on nongovernmental organization, unpublished Report. - Yemen mental health association-YMHA (2007): The Association first Report of the Mental Health in Yemen, YMHA newspaper, vol. 30, 2007, Aden-Yeme. |
| **^±^** Includes:   - World Health Organization. Mental Health Determinants and Populations Team. (‎2001)‎. Atlas of mental health resources in the world 2001. World Health Organization. <https://iris.who.int/handle/10665/66910> - World Health Organization. Mental Health Determinants and Populations Team. (‎2005)‎. Mental health atlas: 2005, Rev. ed. World Health Organization. <https://iris.who.int/handle/10665/43230> - WHO Regional Office for the Eastern Mediterranean Mental health in the Eastern Mediterranean Region: reaching the unreached / WHO Regional Office for the Eastern Mediterranean. p. (WHO Regional Publications, Eastern Mediterranean Series; 29), 2006. - World Health Organization. Regional Office for the Eastern Mediterranean Mental health atlas 2011: resources for mental health in the Eastern Mediterranean Region / World Health Organization. Regional Office for the Eastern Mediterranean, 2013. - Mental health atlas 2014: resources for mental health in the Eastern Mediterranean Region / World Health Organization. Regional Office for the Eastern Mediterranean, EMRO Technical Publications Series, 2016. - Mental health atlas 2017 - Resources for mental health in the Eastern Mediterranean Region. Cairo: WHO Regional Office for the Eastern Mediterranean; 2019. Licence: CC BY-NC-SA 3.0 IGO. - Mental health atlas 2020: review of the Eastern Mediterranean Region Cairo: WHO Regional Office for the Eastern Mediterranean; 2022. Licence: CC BYNC-SA 3.0 IGO. - Also the “country profile” documents for the different years in the countries of the region, available online.   ^*^ Includes:   - Okasha, A. and Karam, E. (1998), Mental health services and research in the Arab world. Acta Psychiatrica Scandinavica, 98: 406-413. - Okasha, A. Mental Health services in the Arab world. Eastern Mediterranean Health Journal, Vol. 5, No.2, 1999. - Okasha A, Karam E, Okasha T. Mental health services in the Arab world. World Psychiatry. 2012 Feb;11(1):52-4. | | | | |

Gift of
